# Increased microbial expression of organic nitrogen cycling genes in long-term warmed grassland soils

**DOI:** 10.1038/s43705-021-00073-5

**Published:** 2021-11-25

**Authors:** Joana Séneca, Andrea Söllinger, Craig W. Herbold, Petra Pjevac, Judith Prommer, Erik Verbruggen, Bjarni D. Sigurdsson, Josep Peñuelas, Ivan A. Janssens, Tim Urich, Alexander T. Tveit, Andreas Richter

**Affiliations:** 1 Centre for Microbiology and Environmental Systems Science, University of Vienna, Vienna, Austria; 2 Department of Arctic and Marine Biology, UiT, The Arctic University of Norway, Tromsø, Norway; 3 Joint Microbiome Facility of the Medical University of Vienna and the University of Vienna, Vienna, Austria; 4 Research Group PLECO, Department of Biology, University of Antwerp, Antwerp, Belgium; 5 Agricultural University of Iceland, Borgarnes, Iceland; 6 CSIC, Global Ecology Unit CREAF- CSIC-UAB, Bellaterra, Catalonia Spain; 7 Department of Bacterial Physiology, University of Greifswald, Greifswald, Germany; 8 International Institute for Applied Systems Analysis, Laxenburg, Austria; 9 Austrian Polar Research Institute, Vienna, Austria

**Keywords:** Climate change, Microbial ecology, Environmental microbiology

## Abstract

Global warming increases soil temperatures and promotes faster growth and turnover of soil microbial communities. As microbial cell walls contain a high proportion of organic nitrogen, a higher turnover rate of microbes should also be reflected in an accelerated organic nitrogen cycling in soil. We used a metatranscriptomics and metagenomics approach to demonstrate that the relative transcription level of genes encoding enzymes involved in the extracellular depolymerization of high-molecular-weight organic nitrogen was higher in medium-term (8 years) and long-term (>50 years) warmed soils than in ambient soils. This was mainly driven by increased levels of transcripts coding for enzymes involved in the degradation of microbial cell walls and proteins. Additionally, higher transcription levels for chitin, nucleic acid, and peptidoglycan degrading enzymes were found in long-term warmed soils. We conclude that an acceleration in microbial turnover under warming is coupled to higher investments in N acquisition enzymes, particularly those involved in the breakdown and recycling of microbial residues, in comparison with ambient conditions.

## Introduction

Soil organic matter (SOM) represents the largest global reservoir of organic carbon (C) and nitrogen (N), and its turnover plays a critical role in global element cycling and the climate system [1]. Rising global temperatures are expected to accelerate the rates of SOM decomposition by accelerating microbial activity and increased expression of enzymes that are responsible for the extracellular depolymerization of high-molecular-weight organic matter, potentially resulting in large releases of greenhouse gases such as CO_2_ into the atmosphere and a positive feedback to climate change [2, 3]. While the processes underlying C dynamics and their repercussion on climate change have been extensively studied, soil N storage and the fate of organic N have received far less attention, despite their intrinsic link to organic C and common dependence on temperature.

Soil microorganisms play a major role in terrestrial N cycling [4, 5], and studies have shown that N accessibility for microorganisms may affect the degradation and turnover rates of the different SOM pools [6, 7]. Furthermore, organic N compounds also contribute substantially to the organic C fraction [7] and microorganisms may decompose organic N to meet their demand in C and energy, rather than their N demand [8]; additionally, the availability of N has been reported to stimulate C-mobilizing enzymes [9].

Organic N in soil consists of high-molecular-weight compounds, such as intracellular and cell-wall bound proteins and nucleic acids of microbial and plant origin, but the great majority of organic N is found in microbial residues (dead microbial cell walls) and its components, such as peptidoglycans, chitin, and cell-wall bound proteins, which may represent up to 80% of the organic N pool [10–12]. The first step in the depolymerization of soil organic N therefore requires the microbial secretion of extracellular enzymes that deconstruct organic N polymers into oligomers or monomers that are small enough to be taken up directly by soil microorganisms and plants and can be further mineralized and incorporated as ammonium (NH_4_^+^) or nitrate (NO_3_^−^). While microbially-mediated inorganic N transformations have been extensively studied [13] we know little of how organic nitrogen cycling is controlled in the environment.

All enzyme-mediated reactions are intrinsically temperature sensitive and warming often causes higher metabolic and turnover rates, as enzymes converge towards their temperature optimum. At the same time, sustained warming also depletes soils of easily accessible organic substrates, leading to reduced microbial biomass levels [14, 15]. In such nutrient-depleted environments, it is even more crucial for organisms to tightly regulate their resource acquisition strategies. The ability to degrade organic N forms is widespread among soil microorganisms [16–18], and substrate concentration and availability are important factors that regulate the production of extracellular enzymes [19, 20]. Under ammonia-limiting conditions, pure culture studies reported higher synthesis rates of proteins involved in the scavenging of alternative N sources as well as in the turnover of proteins [21, 22]. Similarly, increased transcription of genes encoding high-affinity ammonium transporters under N starvation conditions have also been observed [23, 24].

Physiological adaptations of microorganisms to environmental perturbations entail changes in gene expression patterns, and a better understanding on how these regulatory mechanisms respond to the lower organic matter availability caused by warming is urgently needed. Most of our knowledge of ecosystem function comes from cultivation-based studies, enzymatic assays, and/or functional gene amplification studies that target specific processes such as methanogenesis, nitrification and N fixation [25, 26]. However, none of these methods provide a holistic approach to study the functionally active potential of complex microbial communities in the environment. With the emergence of metagenomic and metatranscriptomic approaches we now have powerful tools at hand to study such processes in complex environmental settings in a global change context [27–30].

Here, we combined metatranscriptomics and metagenomics to determine if warming causes a potential acceleration in organic N cycling in soil. We hypothesized that long-term soil warming would lead to persistent transcriptional changes in microbial organic N cycling, specifically higher transcription levels of genes associated with the production of extracellular enzymes involved the depolymerization of organic N from microbial necromass (i.e., of proteins, nucleic acids, chitin, and other organic N-containing substances). Such enzymes would target microbial residues, which become relatively enriched in warmed soils after more labile sources are consumed, and thus constitute alternative sources of both N and C that are readily replenished [11, 31].

We used a geothermal warming experiment in a subarctic grassland in Iceland [32] that provides stable and continuous gradients of soil warming of different durations [33]. The experiment was thus ideal to evaluate possible transcriptional responses to soil warming in situ. Previous studies at these locations reported significant losses of about 40% of soil organic C and N with increased soil warming in the top soil, as well as a decrease in microbial biomass and RNA content [32, 34]. However, biomass-specific microbial growth and turnover rates were consistently higher, even after more than 50 years of warming [14]. Warming did not affect above- or belowground plant biomass or the soil C:N ratio, indicating proportional losses of C and N [14, 15, 35]. A parallel metatranscriptomic study on the potentially active microbial communities from the same set of samples assigned 93–95% of total putative mRNA reads to bacteria [34]. Although there are some known biases towards this group in public databases and extraction methods which require careful interpretation, these data suggest a potentially significant role of bacteria in maintaining the main metabolic pathways detected [34].

## Methods

### Sampling sites and soil sampling

Soil samples were collected from two Icelandic grassland sites subjected to either long-term (>50 years) or medium-term (8 years) geothermal warming from the ForHot natural soil warming experimental site (64° 0′ N, 21° 11′ W). This study site was established in 2012 and comprises replicated transects (2 × 2 m soil plots) representative of different temperature regimes. For each temperature regime, physicochemical parameters such as mean daily soil temperature, mean annual precipitation and soil pH are available [33]. In mid-July 2016 we sampled 4 biological replicates of “Ambient” and “Warmed” (+6 °C above ambient) soil plots from both grasslands to a total of 16 samples (Fig. S1). At the time of sampling the mean ambient temperature of the medium-term warmed (MTW) and LTW soil plots were 13.2 °C and 12.8 °C respectively. The mean elevated temperature of the warmed plots was 22.5 °C and 21.8 °C. From the 16 collected samples, 4 samples representative of the 4 different conditions (i.e. LTW ambient, LTW warmed, MTW ambient, MTW warmed) were selected for metagenome sequencing (Fig. S1). Five subsamples of each soil plot were taken, mixed in a bag, sieved through a 2 mm filter sieve and immediately frozen in liquid nitrogen. Contextual soil data for this specific sampling as well as nutrient pools and potential extracellular leucine aminopeptidase and ß-1,4-N-acetylglucosaminidase activities were collected and measured shortly after sampling (Supplementary Material and Methods; [33, 34]).

### Nucleic acid extraction and sequencing

Total nucleic acids were extracted from 0.3 g of soil from three technical replicates of each biological replicate (total = 48) by bead-beating in the presence of cetyl-trimethylammonium bromide (CTAB) buffer and phenol [36]. Extracts were split and treated with DNase (RQ1, Promega) or RNase A (Thermo Fisher Scientific) before metatranscriptome (*n* = 16) and metagenome (*n* = 4) sequencing, respectively. ﻿ The quality and quantity of DNA and RNA were assessed with a Bioanalyzer (Agilent Technologies, Santa Clara, CA, USA) and the Quant-iT^TM^ PicoGreen^®^ and RiboGreen^®^ kits (Thermo Fisher Scientific), respectively. The absence of DNA in the RNA extracts was verified by PCR assays targeting bacterial SSU rRNA genes. A total of 100 ng RNA was linearly amplified using the MessageAmp II-Bacteria Kit (Ambion Life Technologies) according to the manufacturer’s instructions and an amplification step of 14 h. Metatranscriptomic sequencing libraries were generated using the NEBNext^®^ Ultra RNA Library Prep Kit for Illumina^®^ and sequenced an Illumina HiSeq2500 (v2 chemistry, 2 × 125 bp mode) at the Vienna Biocenter Core Facilities, Vienna, Austria. We used the same filtered metatranscriptomic reads as the ones described in [34]. Shortly, transcripts were quality filtered and trimmed as described in [34] and putative paired-end mRNA reads with at least one hit against the NCBI nr database as of September 2018 were kept for analysis (Table S1).

Metagenome sequencing libraries were generated using the NEBNext Ultra FS II DNA Library Prep Kit for Illumina (New England BioLabs) at the Joint Microbiome Facility of the Medical University of Vienna and the University of Vienna and sequenced on a HiSeq4000 with the HiSeq 3000/4000 SBS Kit (300 cycles) in 2 × 150 bp mode. Metagenomic paired-end reads were screened for residual adapters and PhiX, quality filtered, trimmed and merged using BBtools (BBmap package v. 33.57 http://sourceforge.net/projects/bbmap/) with parameters: ktrim = r k = 21 mink = 11 hdist = 2 minlen = 120 qtrim = r trimq = 15. Following quality filtering, reads were assembled using SPAdes (v3.11.1) with the following options: -m 2000 - only-assembler -k 21,31,121, –meta -t 32. Contigs with less than 1Kb were discarded. Metagenomic read processing and assembly statistics can be found in (Tables S2 and S3). Genes were predicted on assembled metagenomic contigs for each sample (*n* = 4) using Prodigal v2.6.3 [37] with the metagenome (-p) option. Metatranscriptomic read mapping was done using BBmap against the metagenomic genes using default settings (Table S1). The mapping was done against metagenomic instead of metatranscriptomic assemblies because most of the signal peptides that target a protein for secretion are present on N-terminus of the sequence, which is often fragmented and consequently underestimated during metatranscriptomic assemblies. Fragment counts for each gene were converted to fragment per kilobase (FPK) values by dividing the number of fragments mapping to each gene by the length of the gene (in kb). Outlier genes from each dataset with excessively high FPK values were determined by fitting FPK values into a continuous power law cumulative distribution function using the commands available in the powerRlaw R package [38]. Nonzero FPK values per dataset were used to estimate the Xmin parameter with the commands *conpl$new()* and *estimate_xmin()*. Regression between log-transformed FPK values above Xmin and log-transformed rank was performed using the command *lm()* from the base stats package in R. The influence of each FPK on the resulting regression was assessed with the command *influence.measures()* from the base stats package in R. Genes with FPK values that were flagged as “influential” by all six available diagnostics (dfbetas, dffits, covratio, cooks.distance, rstandard and rstudent) and positive residuals (excessively high) were flagged as outliers. The outlier genes that were identified considering each dataset independently were then removed from all datasets and the procedure was repeated until no further outlier genes could be identified. In total, 22 genes were removed using this procedure. A count matrix containing the number of remaining mapped transcripts per metagenomic gene was normalized into a transcript per million (TPM) matrix, taking into account the gene length and the metatranscriptomic library size.

All computations were performed using the CUBE computational resources, University of Vienna, Austria, or were run on the high-performance computing resource STALLO at the University of Tromsø, Norway.

### Identification and screening of genes encoding putatively secreted organic N degradation-related proteins

We first aimed to build a metagenomic backbone representative of all genes at the sampling sites, against which putative mRNA reads could be mapped. A collection of non-redundant metagenomic genes were screened for the presence of protein sequences of interest using Pfam hmm models (*n* = 253) and *hmmsearch* with the trusted cut-off of each individual model [39, 40]. We included known protein domains present in enzymes involved in peptidoglycan and chitin catabolism (e.g., glycosyl hydrolases from families 18, 19, 20, 22), as well as those involved in nucleic acid degradation (e.g., endo/exonucleases) and those involved in protein catabolism (e.g., metallo- and serine peptidases) (Table S4). We additionally included extracellular domains such as the LysM and LTD domains, which are found in a wide range of carbohydrate-targeting extracellular proteins. Only hits whose reverse search against the Pfam database (v.32) using *hmmscan* returned the target model as best hit were considered further. Hits with more than one attributed Pfam domain were individually checked, and only the one with the highest bitscore was kept. This list of candidate genes was further screened to identify conserved domains signaling putatively extracellular secretion: SignalP (v 4.1) was used to search for signal peptides, with the default settings for eukaryotes, Gram-negative bacteria and Gram-positive bacteria [41]. SecretomeP (v 2.0) was used to screen for alternatively secreted proteins in Gram-negative and Gram-positive bacteria with default settings [42]. Only proteins that had a SecP score above 0.5 and that simultaneously did not contain a signal peptide were considered further. Finally, predicted protein subcellular localization was inferred using Psort (v 3.0) with the default settings from archaea, Gram-negative, and Gram-positive bacteria [43]. Proteins whose location was set as “Extracellular”, “Outer Membrane” and “Cell Wall” were considered further. We used these three approaches in order to circumvent limitations associated with the performance of each software in identifying different secretion pathways [41]. A total of 15409 metagenomic genes met the above-mentioned criteria and were subsequently annotated against an updated database containing predicted proteins from all protist, fungal, bacterial, and archaeal genomes and MAGs in the JGI and NCBI [44]. The database also included all fungal genomes from the NCBI RefSeq database. The total number of predicted proteins in this database was 73.4 million, as of March 2020. Additionally, genes were annotated against the CAZy V.9 database [45] as well as the NCBI nr protein database. Our criteria to identify homologs against these databases using BLASTP were an amino acid identity above 40%, bit scores above 50, a subject coverage of at least 30%, a minimum alignment length of 50 residues, and the –more-sensitive option on Diamond [46]. We excluded 1360 genes with no hits on the genomic database. In addition, we manually excluded 1910 genes that were not involved in organic N degradation, but still contained relevant protein family domains and had been flagged as encoding putatively secreted proteins. This was the case for chaperone and precursor proteins, as well as proteins involved in cell division, that might be secreted but not actively involved in the hydrolysis of organic N compounds. The final dataset consisted of 12012 genes encoding putatively secreted proteins involved in organic N cycling, which could be assigned to 144 Pfams (Table S5). We additionally used the functional annotation to better define the target substrate of proteins encoded by particular genes. This was particularly important for genes encoding for proteins containing extracellular domains such as LysM or LTD. For example, the LTD domain can be present in proteins that target either nucleic acids or proteinaceous compounds. Based on the annotation of genes containing this domain, we were able to either assign a preferred substrate, or exclude other target substrates. Finally, genes of interest were taxonomically classified using the LCA algorithm based on the NCBI taxonomy implemented in Diamond (--outfmt 102, top = 5, min_id = 40).

### Data analysis

All statistical analyses were performed using R v3.6.1. Previously identified genes involved in extracellular organic N degradation were extracted from the main TPM matrix, in order to keep relative abundance expression levels in relation to the remaining genes. The table was filtered in order to keep only genes with mapped transcripts (n = 9413), which comprised 131 Pfams. We applied a function filter, by first aggregating the TPM matrix Pfams, and then keeping only the domains present in all the four biological replicates of at least one group (Table S6). We additionally performed the function filter on each grassland site individually (Table S6).

To filter the functions that showed similar responses under warming irrespective of warming duration, we aggregated the TPM matrix by their Pfam annotation and considered a Pfam to be more abundant under warmed conditions if the mean TPM abundance across the four biological replicates was higher under warming than under ambient conditions. The statistical significance of this trend was then assessed by parametric or nonparametric tests according to whether parametric conditions (normal distributions and homogeneity of variances) were met or not (Table S7). *P*-values were adjusted for the false-discovery rate using the Benjamin–Hochberg correction method (*p.adjust*(method = “BH”)).

A nonmetric multidimensional scaling (NMDS) was performed with function *metaMDS()* from the R package vegan [47] to assess the effect of temperature and warming duration on the transcribed organic N-related functions. A principal component analysis (PCA) was performed with function *PCA()* from the FactoMineR [48] R package to investigate the effect of temperature and warming duration on the physicochemical soil properties and cumulative relative transcription levels of key genes encoding putatively secreted organic N-degrading enzymes. The cumulative sum of relative transcriptional abundances of key genes was calculated by adding up the TPM values of all genes associated with the degradation of a particular substrate, which yielded four values representative of the four organic N substrates under study.

The *adonis()* function was used to perform permutational analyses of variance (PERMANOVA) and assess the effect of temperature and warming duration on the observed functional and taxonomic abundance patterns. Heatmaps were generated using the *pheatmap()* function of the pheatmap R [49] package. Rows were clustered using complete linkage, according to similar distribution patterns. All dissimilarity matrices used were generated using the Canberra distance, and PERMANOVAs were ran with 9999 permutations. The number of unique genes and functions was calculated by iterative (*n* = 10) rarefaction with replacement at a fixed depth (75% of the reads of the sample with the lowest number of total genes), using the number of transcripts that mapped to each gene potentially involved in the degradation of organic N sources as a probability vector. The function *rmultinom()* from the R stats package was used, and a rounded mean of the multinomial probabilities was used to compute gene and function richness values using the function *richness()* from the microbiome R package. The same approach was used to calculate the number of unique taxonomic groups.

## Results

### Common responses to soil warming

We obtained soil metatranscriptomes of four biological replicates of previously characterized locations representing ambient and warmed (+6 °C) conditions in two grasslands subjected to 8 and > 50 years of warming. We additionally obtained four metagenomes representative of each condition. Metatranscriptomic reads were mapped to predicted metagenomic genes in order to identify transcriptional differences in organic N degradation processes in response to warming.

In both grasslands, we obtained a total of 9074 unique metagenomic open reading frames (ORFs), representing predicted genes and contained 106 different protein family domains (Pfams) associated with organic N degradation processes. Most of the genes were affiliated with bacteria (85.1 and 86.7% in the MTW and long-term warmed (LTW) grasslands, respectively), whereas eukaryotic and archaeal genes represented approximately 0.01% of these genes. The remaining fraction of the identified genes could not be phylogenetically classified at the domain level. After excluding such genes and those assigned to “Unclassified Bacteria”, 75% the genes had a phylum-level classification, 40% had a class-level classification and 19.3% had an order-level classification. Genes phylogenetically associated with phylum Proteobacteria were the most abundant in both grasslands (29–32%), followed by Acidobacteria (21–23%) and Actinobacteria (7.2–8.2%). We found no significant differences in the number of unique taxonomic groups between ambient and warmed soils regardless of the warming duration (Fig. S2).

**Fig. 1:**
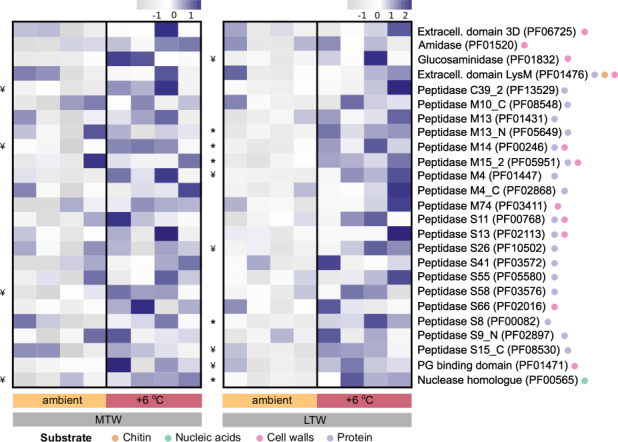
Shared transcriptional responses to warming. Shared protein family domains (Pfams) associated with organic N degradation that had higher mean relative transcript abundances under warming than ambient conditions (see Methods for details). Each heatmap is normalized by row (z score). Biological replicates are ordered by their temperature profile, and the potential target substrates of each Pfam are indicated by the colored circles after the family domain name. Significant differences between ambient and warmed conditions for each Pfam were assessed by parametric or nonparametric *t*-tests (two-tailed), depending on whether or not the variable met parametric assumptions. All *p* values were adjusted for the false-discovery rate (Benjamin-Hochberg correction). Adjusted significant *p* values < 0.05 are indicated on the left of each heatmap by *, and *p* values that became nonsignificant after correction are indicated by ¥. The results of the full statistical analysis and exact *p*-values are presented in Table S7. PG peptidoglycan; Peptidase S_ serine peptidase; Peptidase M_ metallopeptidase; Peptidase C_ cysteine peptidase; LTW Long-term warmed grassland; MTW Medium-term warmed grassland.

Approximately 55% of the Pfams involved in organic N degradation identified in each grassland showed higher mean transcription levels in the warmed plots (LTW, 60 out of 104 Pfams; MTW, 50 out of 94 Pfams). Twenty-five Pfams were shared between both grasslands (i.e. showed the same response, irrespective of warming duration), and most were classified as metallopeptidases (*n* = 8) and serine peptidases (*n* = 10) or had domains involved in peptidoglycan catabolism (*n* = 5) (Fig. 1).

### Warming influences physicochemical properties and transcription of genes encoding organic N-degrading enzymes

**Fig. 2:**
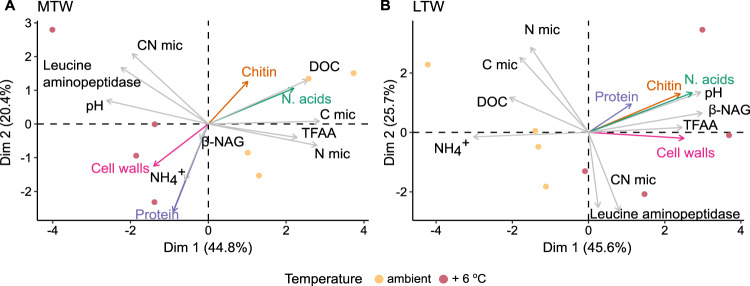
Principal component analysis (PCA) depicting the effect of warming on soil physicochemical properties and relative transcription of genes involved in the degradation of organic N. Warming effect on soil physicochemical properties and the cumulative relative transcription level of genes encoding putative organic N-degrading enzymes are shown for the medium-term warmed (MTW) (**A**) and long-term warmed (LTW) (**B**) grasslands. DOC, dissolved organic (µg C g^−1^ dry weight soil); C mic, microbial biomass C (µg C g^−1^ dry weight soil); N mic, microbial biomass N (µg N g^−1^ dry weight soil); TFAA, total free amino acids (µg N g^−1^ dry weight soil); CN mic, microbial C to N ratio. DOC, pH, and microbial biomass C and N contents for these samples were retrieved from [34]. Enzyme data are expressed per unit of microbial biomass C (Table S8). The cumulative relative transcription of genes involved the degradation of the different organic N compounds is indicated by the colored arrows: chitin (orange); nucleic acids (green); cell walls (pink); protein (purple).

A PCA was used to examine the composition of soils with respect to physicochemical characteristics and cumulative sum of relative transcriptional abundances of key genes encoding putatively secreted organic N-degrading enzymes. Both axes captured between 66.2 and 73% of the variation in the MTW and LTW grassland respectively, and ambient and warmed soils showed a clear separation along the first dimension (Fig. 2; Fig. S3).

Warmed MTW grassland plots were characterized by non-significantly higher microbial CN ratios, leucine aminopeptidase potential activity, and higher relative transcription levels of genes encoding enzymes involved in the degradation of cell walls and proteins (Fig. S4; Table S7). Ambient plots were characterized by lower levels of organic C (DOC), total free amino acids (TFAA), and microbial biomass C and N [34]. In addition, genes encoding enzymes putatively involved in the degradation of chitin and nucleic acids also showed higher relative transcription levels under ambient conditions. In the LTW grassland, warmed plots were characterized by higher relative transcription of genes coding for enzymes involved in the degradation of all types of organic N sources, as well as higher potential enzymatic activities and microbial CN ratios (Fig. S4; Tables S7 and S8). Similarly to the MTW, the ambient plots of the LTW grasslands were characterized by higher microbial biomass, DOC and TFAA levels [34] (Fig. 2B; Fig. S4).

### Warming leads to an upregulation of organic N-degrading genes and enzyme families

**Fig. 3:**
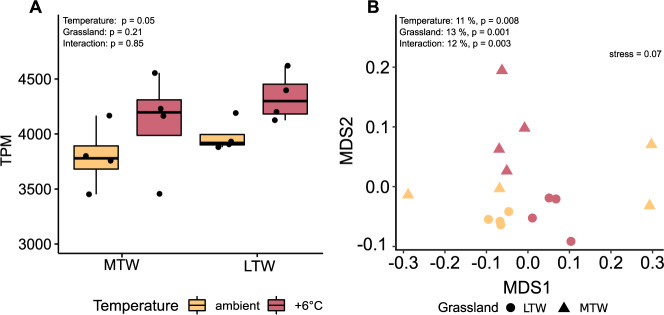
Differences in relative transcription levels of genes involved in organic N degradation. The relative transcription levels of all genes encoding secreted proteins putatively involved in organic N degradation in relation to all protein-coding genes in each sample are shown as transcripts per million (TPM) between ambient and warmed soil plots of the medium-term warmed grassland (MTW) and the long-term warmed grassland (LTW) (**A**). Inside the boxplots, the median value is shown. A two-way ANOVA was used to assess statistical the statistical significance. Subplot (**B**) shows a nonmetric multidimensional scaling (NMDS) ordination of all samples, according to the transcribed functions under analysis. The percentage of variation explained by each factor in a PERMANOVA is shown in the NMDS plot. Unpaired *t*-tests (two-tailed) were used after checking the parametric assumptions, and *p*-values were adjusted for the false-discovery rate (Benjamin-Hochberg correction). Detailed statistical results can be found in Table S7.

Warming resulted in significantly higher relative transcription levels of genes encoding putatively secreted proteins involved in organic N degradation in comparison with ambient plots regardless of warming duration (Fig. 3). In addition, a higher variability in the relative expression of genes encoding for secreted proteins putatively involved in organic N degradation was observed in the metatranscriptomes of the MTW grassland compared to the LTW grassland (3526 TPMs ± 227 and 3939 TPMs ± 77, in MTW and LTW grasslands respectively; Fig. S5B). The number of unique transcribed genes or functions also did not differ significantly between the ambient and warmed conditions (Fig. S6; Table S7).

Additional multivariate analyses (PERMANOVA) showed that the transcribed Pfams were significantly affected by temperature, warming duration and an interaction of the two (Fig. 3B). However, the dispersion between groups was not homogeneous (Fig. S7; Permdist, *p* < 0.05), an issue likely raised by the high group variance dispersion of ambient MTW plots. This meant that the PERMANOVA assumptions were not met, and results should be interpreted with care.

### Individual grassland transcriptional responses to warming

We observed a significantly different community structure of the microbial taxa transcribing the genes of interest in response to warming in the LTW grassland (PERMANOVA, *F* = 5.17, *R*^2^ = 0.46, *p* = 0.03) (Fig. S8B) in comparison with ambient soil plots. Warmed LTW grassland soils were characterized by higher transcription levels of genes affiliated with Planctomycetes, Beta- and Deltaproteobacteria, whereas in ambient soils, the transcription levels of Actinobacteria, Alpha- and Gammaproteobacteria were higher (Fig. S8B).

**Fig. 4:**
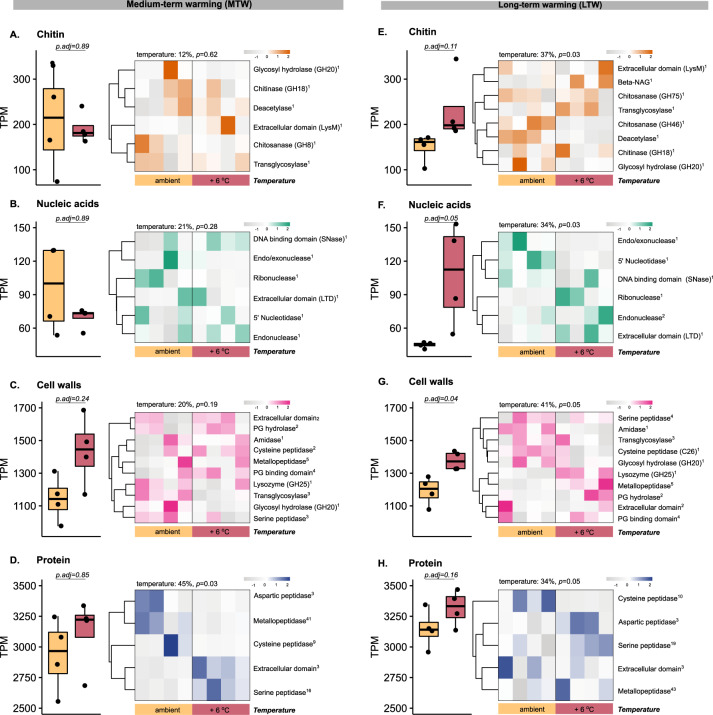
Grassland-specific transcriptional responses to warming. Warming-induced differential transcription levels, in transcripts per million (TPM), of genes and enzyme families involved in the degradation of chitin (**A**, **E**), nucleic acids (**B**, **F**), cell walls (**C**, **G**) and proteins (**D**, **H**) in the MTW and LTW grasslands. The boxplots depict an aggregated sum of transcripts mapped to genes assigned to the degradation of a particular substrate relative to all transcripts, according to ambient or warmed (+6 °C) conditions. Significant differences between ambient and warmed conditions for each substrate were assessed by parametric or nonparametric *t*-tests, depending on whether or not the variable met the assumptions (Table S7). All *p* values were adjusted for the false-discovery rate (Benjamin–Hochberg correction). Heatmaps were built using TPM normalized count matrices for each substrate and depict the different functional domains (Pfams) assigned to a substrate. The data were normalized by row (z score) and clustered using complete linkage, according to similar abundance patterns. The superscripted numbers indicate the number of Pfams aggregated in each category. The percentage of variation explained by temperature is shown on top of the heatmaps (PERMANOVA analysis).

We also found higher transcription levels and a shift in the transcriptional structure of genes encoding enzymes potentially involved in the degradation of chitin in response to warming (Fig. 4E). Warmed LTW grassland plots were characterized by higher transcription of genes annotated as β-N-acetylglucosaminidases, chitosanases and transglycolases (Fig. 4E), but the chitin-degrading microbial community structure was not significantly affected by warming (Fig. S9A). Genes encoding proteins potentially involved in nucleic acid degradation also showed significantly higher transcription levels under warming in the LTW grassland in comparison with ambient conditions (Fig. 4F). Similarly, there were also significant changes in the relative transcription levels of genes encoding the different protein family domains associated with nucleic acid degradation. These differences also entailed taxonomic changes in the nucleic-acid-degrading microbial community between ambient and warmed plots (Fig. S9B). Warmed LTW grassland plots were characterized by higher transcription levels of genes affiliated with Betaproteobacteria, *Candidatus* phylum Rokubacteria, Deltaproteobacteria, and Verrucomicrobia (Fig. S9B), transcribing genes annotated as extracellular ribonucleases, endonucleases, and extracellular domains (Fig. 4F). The significantly higher relative transcription levels of genes potentially encoding proteins involved in cell wall degradation under warming were characterized by higher relative transcription levels of peptidoglycan hydrolases such as lysozymes, metallo- and serine peptidases, as well as several peptidoglycan-binding domains (Fig. 4G). Significant differences in the community structure of taxa transcribing genes encoding these enzymes were characterized by higher relative contributions of Alpha- and Deltaproteobacteria, Acidobacteria and *Candidatus* Rokubacteria under warming. (Fig. S9C). Notably, no significant effect of warming was found regarding the transcription levels of genes associated with protein degradation (Fig. 4H). Nevertheless, temperature significantly affected the structure of protein-degrading family domains, as indicated by higher transcription levels of genes annotated as aspartic, metallo-, and serine peptidases in the warmed plots. These functions were primarily associated with Planctomycetes, Acidobacteria and Betaproteobacteria (Fig. S5D).

The number of significant differences between warmed and ambient soils were fewer in the MTW grassland than in the LTW grassland (Fig. 4A; Figs. S8B and S10). However, functions associated with cell wall and protein degradation showed trends toward higher relative transcription levels under warming, similarly to what was observed in the LTW grassland (Fig. 4). Medium-term warming also resulted in significantly different functional changes of genes encoding enzymes involved in protein degradation, which were characterized by higher transcription of genes encoding serine peptidases and extracellular domains (Fig. 4D).

## Discussion

The depolymerization of complex organic N compounds to oligomers or monomers is considered to be the rate-limiting step in terrestrial N cycling [50–52]. Organic N compounds are also valuable sources of C and energy [8], and whenever they are scavenged for C or energy rather than N, microorganisms may excrete the excess N as ammonium, fueling inorganic N transformations. Studies have suggested that the main determinants of organic N depolymerization rates are substrate availability and accessibility, as well as the activity of depolymerizing enzymes of microbial origin [50, 53]. Warming directly accelerates decomposition processes by soil microorganisms, microbial activity as well as the growth and turnover of microbial communities, contributing to a gradual depletion of substrates [3, 32]. Lower microbial biomass in response to substrate limitation, coupled with higher mass-specific microbial activity, results in reduced microbial C and N immobilization and in consequent C and N losses from the system with potential positive feedbacks to climate change [14, 15, 54].

Increasing metabolic investment towards the production of depolymerizing enzymes that act on insoluble high-molecular-weight plant and SOM sources is therefore a likely key strategy to overcome the limitation of accessible nutrients [21, 22, 55, 56]. Indeed, we observed higher transcription levels of genes and enzyme families involved in organic N degradation in response to warming, particularly in the LTW grassland (Fig. 4), although we found no significant difference between the MTW and LTW grasslands (Fig. S5). This observation is consistent with a trend towards higher hydrolytic enzymatic activities per unit of microbial biomass that was also observed in response to long-term warming (Fig. S4). Even though measurements of potential enzymatic activities are general indicators, they reflect the activity of a limited set of enzymes under optimal conditions, thus providing an incomplete assessment of what happens under natural conditions.

In our study, most of the detected transcribed genes encoding putatively secreted proteins were associated with the degradation of microbial residues such as cell walls and proteins, consistent with these substrates representing major sources of organic N under warming [11] in line with our hypothesis. We detected significantly higher relative transcription levels of genes encoding extracellular peptidases from family M13, thermolysins from family M4 and serine peptidases from family S8 in LTW soils. These peptidase families are described as having strong extracellular proteolytic activity and to act on proteins and small peptides to sustain bacterial nutrition [16, 57].

Additionally, genes encoding peptidases from families M14 and M15 also showed consistently higher relative transcription levels in the warmed plots of the LTW grassland. Extracellular zinc-containing carboxypeptidases from family M14 are synthesized without signal peptides and are involved in nutrition and in the metabolism of bacterial cell walls [58, 59]. Cell-wall hydrolases from family M15 include zinc-containing D-Ala-D-Ala carboxypeptidases and dipeptidases involved in cell-wall biosynthesis and metabolism [59].

The increased transcription levels of genes encoding these families of extracellular peptidases in the LTW grassland thus suggests that microorganisms exposed to long-term warming allocate more resources into cell wall and protein degradation processes, corroborating sustained higher microbial turnover rates reported previously for LTW soils [14]. Our results are further supported by previous observations that highlighted the importance of microbial residues as potential C and N sources under warming [11, 60].

We detected few to no changes in the structure of the microbial communities responsible for producing the enzyme families under study. This suggests that physiological changes rather than major changes in community composition were responsible for adaptations to soil warming in this subset of the microbial community of our experiment, especially since the ability to produce and secrete these types of enzymes is widespread among soil microorganisms. Nevertheless, the few observed phylogenetic changes between ambient and warmed conditions of the LTW grassland reflected a potentially relevant contribution of Deltaproteobacteria in degrading all organic N forms and of Planctomycetes in degrading proteinaceous compounds. On the other hand, Alpha- and Gammaproteobacteria and Actinobacteria showed higher transcription levels of genes involved in the degradation of all organic N forms under ambient conditions, suggesting the existence of group-specific responses to warming and/or substrate preferences.

Previous observations reported an absolute decrease in fungal phospholipid fatty acid markers under elevated temperatures [32] in comparison to ambient conditions. Supporting this view, we observed higher transcription levels of deacetylases and chitosanases (GH 46) in ambient conditions in the LTW grassland. Glycosyl hydrolases from family 46 known to degrade chitosan, a polymer obtained after the deacetylation of chitin. The higher relative transcription levels of genes encoding deacetylases and chitosanases under ambient conditions coupled to higher fungal PLFA levels suggests that fungal cell walls are contributing more to the microbial necromass under ambient than under warmed conditions and decreasing the importance of chitin as a source of organic matter under warming. While the observed higher relative transcription levels of chitin-degradation genes in the LTW grassland might contradict previous findings, high levels of functional redundancy are common to the degradation of microbial residues such as chitin and peptidoglycan present in microbial cell walls [18, 61], so chitinases may also be involved in the degradation of bacterial cell walls. Another possible explanation is that while fungi are less abundant in warmed soils, their turnover is increased, resulting in a potentially higher contribution of fungal cell-wall material for decomposition. Along these lines, we did not detect changes in the transcription levels of genes containing domains present in classic chitinases (i.e., glycosyl hydrolases from families 18 and 19) in response to warming.

Notably, we detected higher relative transcription levels of genes annotated as β-N-acetylglucosaminidases in the LTW soils, but the transcription of these genes was not even detected in MTW soils. Thus, regarding the LTW grassland, we cannot exclude the possibility that the transcription of genes associated with chitin degradation is also associated the degradation of other substrates present at higher levels or more easily accessible under natural conditions. A methodological limitation of this study is the impossibility of linking a protein family domain directly to the degradation of a particular substrate, given that there might be high levels of enzyme promiscuity (i.e., an enzyme acting on multiple substrates). We tried to account for this limitation by carefully evaluating the functional annotation of each gene encoding a putatively secreted enzyme and assigning it to the degradation of all the substrates that it had the potential to act on. Another limitation of this study is that only one sampling time point for metatranscriptomics was taken. This is particularly relevant, due to the short half-life of mRNA and its associated high temporal variability. However, our results demonstrate similar responses to warming in both grassland ecosystems. Therefore, we believe that the expression patterns we found are robust, even though we cannot draw conclusions about their variability in time.

We were surprised to find few to no transcribed fungal genes associated with organic N degradation in the metatranscriptomes in this study. Saprotrophic fungi are known to produce a wide range of extracellular enzymes that degrade organic N sources [62]. However, the underrepresentation of eukaryotes in metagenomic and metatranscriptomic datasets has been also reported in other studies [63]. We observed an increase in glycosyl hydrolases family 75 under warming in LTW which mostly includes chitosanases of fungal origin. However, all 12 genes that contained this functional domain were assigned to Bacteria and had no hits on the CAZy database, which suggests the presence of a still uncharacterized set of bacterial chitosanases.

In this study, we highlight the potential of metatranscriptomics as a tool that offers a comprehensive overview of the active functional potential for the degradation of organic N under warming. Our results also provide grounds for the formulation of hypotheses that enable further targeted research focused on specific sets of enzymes of interest.

Finally, our study demonstrates that sustained soil warming leads to higher transcription levels of genes and enzyme families involved in the degradation of N-rich polymers, especially those found in microbial necromass. This suggests that the recycling of microbial remains is a key process to support consistently higher growth and turnover rates of microbial communities when labile C and N substrates become limiting, already after 8 years of sustained warming. Considering that (sub)arctic ecosystems are especially vulnerable to climate change, our results provide a mechanistic insight into the micro-scale mechanisms that lead to ecosystem-scale responses to soil warming.

## Supplementary information

Supplementary Material and Methods

Supplementary Figures and Tables

Table S3

Table S4

Table S5

## Data Availability

The metatranscriptomic reads were obtained from [34] and are available at the NCBI Short Read Archive (SRA) under BioProject ID PRJNA663238. The metagenomic raw reads and assemblies obtained in this study can be found under BioProject ID PRJNA746424. The corresponding metadata is available as Supplementary Material.
