# Supplementary material for: Increased microbial expression of organic nitrogen cycling genes in long-term warmed grassland soils
Source: ISME Commun. 2021 Nov 25;1:69. doi: 10.1038/s43705-021-00073-5 (PMC9723740; doi:10.1038/s43705-021-00073-5)
Supplement: Supplementary file 1 — Supplementary Material and Methods [file 43705_2021_73_MOESM1_ESM.docx]

**Supplementary Material and Methods**

**Site description and experimental site**

The study sites are located in southern Iceland and include two grasslands subjected to different geothermal soil warming durations. The MTW grassland became warmed as a result of an earthquake in 2008. The LTW has been subjected to sustained warming for over 50 years and is located 2.0-2.5 km NW of the MTW. Regular field monitoring has been done on the hot spots of the LTW grassland since 2005. The sampling sites from the MTW and LTW grasslands were remapped after the 2008 earthquake and are a part of the Forhot study since 2012 (<https://forhot.is>). The ambient plots of both grasslands are dominated by *Agrostis capillaris* grasses, some herbs and mosses (1). The soil type at both locations is classified as Silandic Andosol and the topsoil C and N concentrations in ambient soils are similar and around 5% and 0.45% respectively. Furthermore, previous data showed that geothermal water did not reach the soil and root zone of any of the warmed plots, meaning that the observed effects are due to increasing temperature alone (1). A full characterization of the study site and all sampling plots and conditions is available in (1). Further soil contextual data for this particular sampling can be found in (2).

**Nitrogen pools and potential enzymatic activity measurements**

﻿Ammonium (NH_4_^+^) concentrations were determined photometrically from 1M KCl extracts of non-fumigated soils (3) and total free amino acids (TFAA) were measured fluorometrically using the OPAME assay (4,5)

Potential extracellular enzyme activities were measured as described in (6). Briefly, one gram of fresh sieved soil was suspended in 100 ml of sodium acetate buffer (100 mM, pH 5.5) and ultrasonicated at low energy(7). Leucine aminopeptidase (EC 3.4.11.1) and ﻿ß-1,4-N-acetylglucosaminidase (EC 3.2.1.30) activities were measured fluorometrically (8). A 200 µl soil suspension and 50 µl of substrate (L-Leucine-7-amido-4-methylcoumarin hydrochloride and 4-Methylumbelliferyl N-acetyl-β-D-glucosaminide) were pipetted into black microtiter plates in triplicates. Methylumbelliferyl (MUF) was used for the calibration of N-acetylglucosaminidase, whereas aminomethylcoumarin (AMC) was used for the calibration of leucine amino-peptidase. Plates were incubated in the dark for 180 minutes and fluorescence was measured at 450 nm emission at an excitation of 365 nm (Tecan Infinite M200 fluorometer, Werfen, Austria).

**References:**

1. Sigurdsson BD, Wallander H, Gunnarsdóttir GE, Richter A, Sigurðsson P, Leblans NIW, et al. 37 Geothermal ecosystems as natural climate change experiments: The ForHot research site in 38 Iceland as a case study. Icelandic Agric Sci. 2016;29:53–71.

2. Söllinger A, Séneca J, Dahl MB , Prommer J, Verbruggen E, Sigurdsson BD, et al. Downregulation 40 of the microbial protein biosynthesis machinery in response to weeks, years and decades of 41 soil warming. 2021 Research Square preprint; DOI: 10.21203/rs.3.rs-132190/v2

3. Hood-Nowotny R, Umana NH-N, Inselbacher E, Oswald- Lachouani P, Wanek W. Alternative Methods for Measuring Inorganic, Organic, and Total Dissolved Nitrogen in Soil. Soil Sci Soc Am J. 2010;74(3):1018–27.

4. Jones DL, Owen AG, Farrar JF. Simple method to enable the high resolution determination of total free amino acids in soil solutions and soil extracts. Soil Biol Biochem. 2002;34(12):1893–902.

5. Prommer J, Wanek W, Hofhansl F, Trojan D, Offre P, Urich T, et al. Biochar Decelerates Soil 49 Organic Nitrogen Cycling but Stimulates Soil Nitrification in a Temperate Arable Field Trial. 50 PLoS One. 2014;9(1):e86388

6. Kaiser C, Koranda M, Kitzler B, Fuchslueger L, Schnecker J, Schweiger P, et al. Belowground carbon allocation by trees drives seasonal patterns of extracellular enzyme activities by altering microbial community composition in a beech forest soil. New Phytol. 2010;187(3):843–58.

7. Stemmer M, Gerzabek MH, Kandeler E. Organic matter and enzyme activity in particle-size fractions of soils obtained after low-energy sonication. Soil Biol Biochem. 1998;30(1):9–17.

8. Marx M-C, Wood M, Jarvis SC. A microplate fluorimetric assay for the study of enzyme diversity in soils. Soil Biol Biochem. 2001;33(12):1633–40.
