# Supplementary material for: Increased microbial expression of organic nitrogen cycling genes in long-term warmed grassland soils
Source: ISME Commun. 2021 Nov 25;1:69. doi: 10.1038/s43705-021-00073-5 (PMC9723740; doi:10.1038/s43705-021-00073-5)
Supplement: Supplementary file 2 — Supplementary Figures and Tables [file 43705_2021_73_MOESM2_ESM.docx]

**Supplementary figures and tables**

**Content**

**Supplementary figures**

Figure S1. Experimental design and sampling setup

Figure S2. Unique taxonomic groups encoding transcribed genes involved in the degradation of organic N.

Figure S3. Effect of warming on soil physicochemical properties and relative transcription of genes involved in the degradation of organic N (n=16)

Figure S4. Physicochemical parameters and enzymatic activities

Figure S5. Number of transcribed ON cycling genes per warming and grassland profile

Figure S6. Number of unique functions and genes

Figure S7. Analysis of multivariate homogeneity of group dispersions (PERMDIST)

Figure S8. Taxonomic classification of all transcribed genes

Figure S9. Taxonomic classification of all transcribed genes parsed by substrate in the LTW grassland

Figure S10. Taxonomic classification of all transcribed genes parsed by substrate in the MTW grassland

**Supplementary tables**

Table S1. Metatranscriptomic/metagenomic read processing and mapping statistics

Table S2. Metagenomic read processing

Table S3. Metagenomic assembly statistics*

Table S4. List of Hidden Markov Models used*

Table S5. Functional annotations of metagenomic encoded genes*

Table S6. Datasets filtering summary

Table S7. Detailed results from multiple testing

Table S8. Nutrient pools and enzymatic data

*submitted as a supplementary Excel document

**Supplementary Figures**


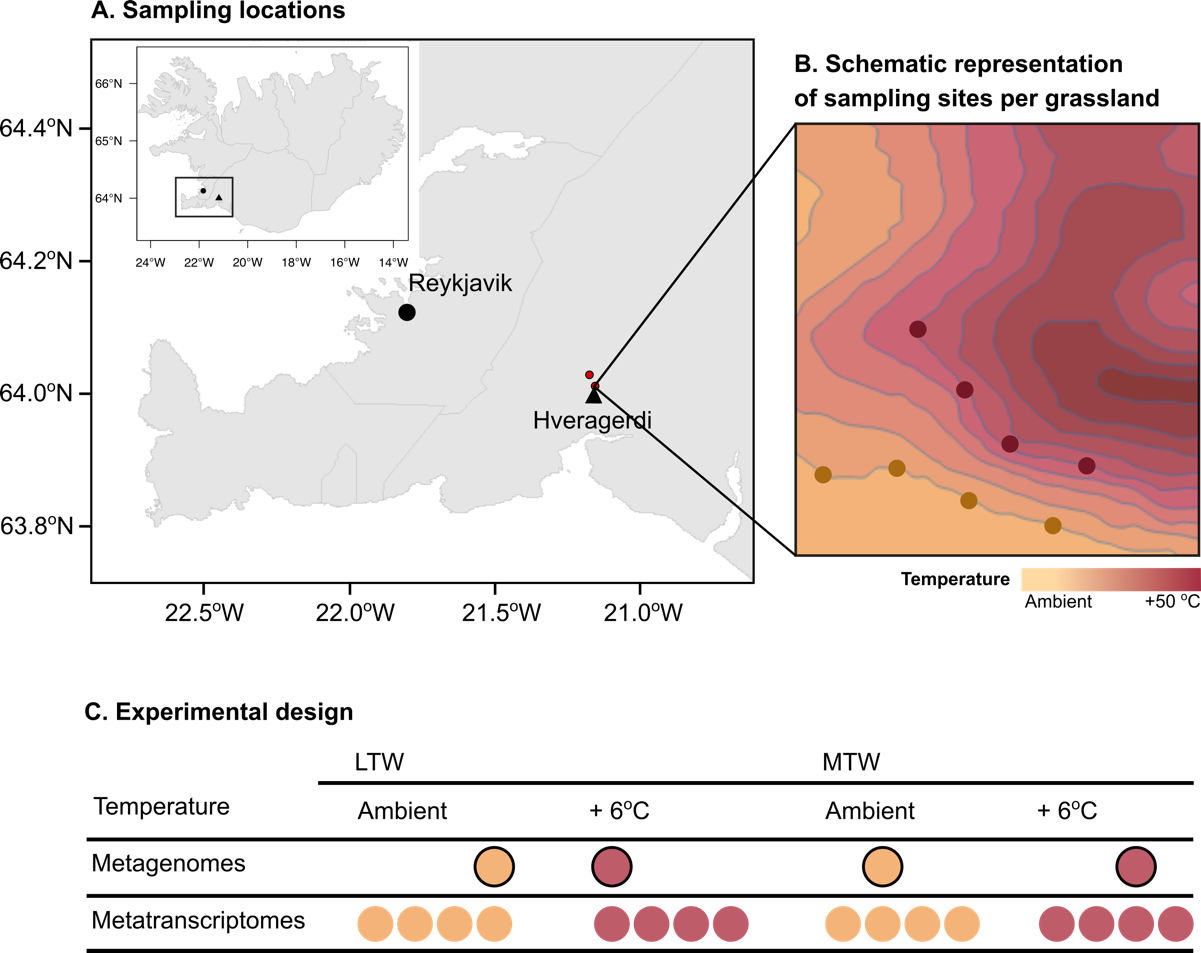


**Figure S1.** **Sampling locations and schematic representation of the experimental design and sampling strategy.** A – Location of the sampling locations in Iceland. The red circles represent the two grasslands; B - Schematic (non-scaled) representation of the sampled sites, depicting four transects sampled in both the long-term warmed and in the medium-term warmed grassland (n=16). The isothermal grey lines represent different temperature profiles, and the yellow and dark red circles represent the temperature profiles sampled in this study. The isothermal color gradient represents an increase in soil temperature, which reflects the increasing proximity to the geothermal heat source. C- Selected experimental design: biological replicates of the same temperature profile were collected, and total nucleic acids were extracted in technical replicates and subsequently pooled. The best representative sample of each condition (depicted in black contoured circles) was chosen for metagenome sequencing. More details on the sample extraction and processing can be found in the Methods section. Further site characterizations can be found in the Supplementary Material and Methods section, Sigurdsson et al. (2016) and https://forhot.is.

**Figure S2. Unique taxonomic groups encoding transcribed genes involved in the degradation of organic N.** Unique classified phyla (A), classes (B) and orders (C) in the medium-term warmed (MTW) and long-term warmed (LTW) grasslands. Genes with an unknown classification at the domain level, and those classified as from “Unclassified” or “Uncultured” bacteria, were excluded. Significant differences between groups were assessed using two-tailed Mann-Whitney tests. Detailed statistics are presented in Table S7.

**Figure S3. Effect of warming on soil physicochemical properties and relative transcription of genes involved in the degradation of organic N (n=16)**

The warming effects on soil physicochemical properties and in the cumulative relative transcription level of genes encoding putative organic N-degrading enzymes are shown (n=16). DOC, dissolved organic (μg C g-1 dry weight soil); C mic, microbial biomass C (μg C g-1 dry weight soil); N mic, microbial biomass N (μg N g-1 dry weight soil); TFAA, total free amino acids (μg N g-1 dry weight soil); CN mic, microbial C to N ratio. DOC, pH and microbial biomass C and N contents for these samples were retrieved from [34]. Enzyme data are expressed per unit of microbial biomass C.


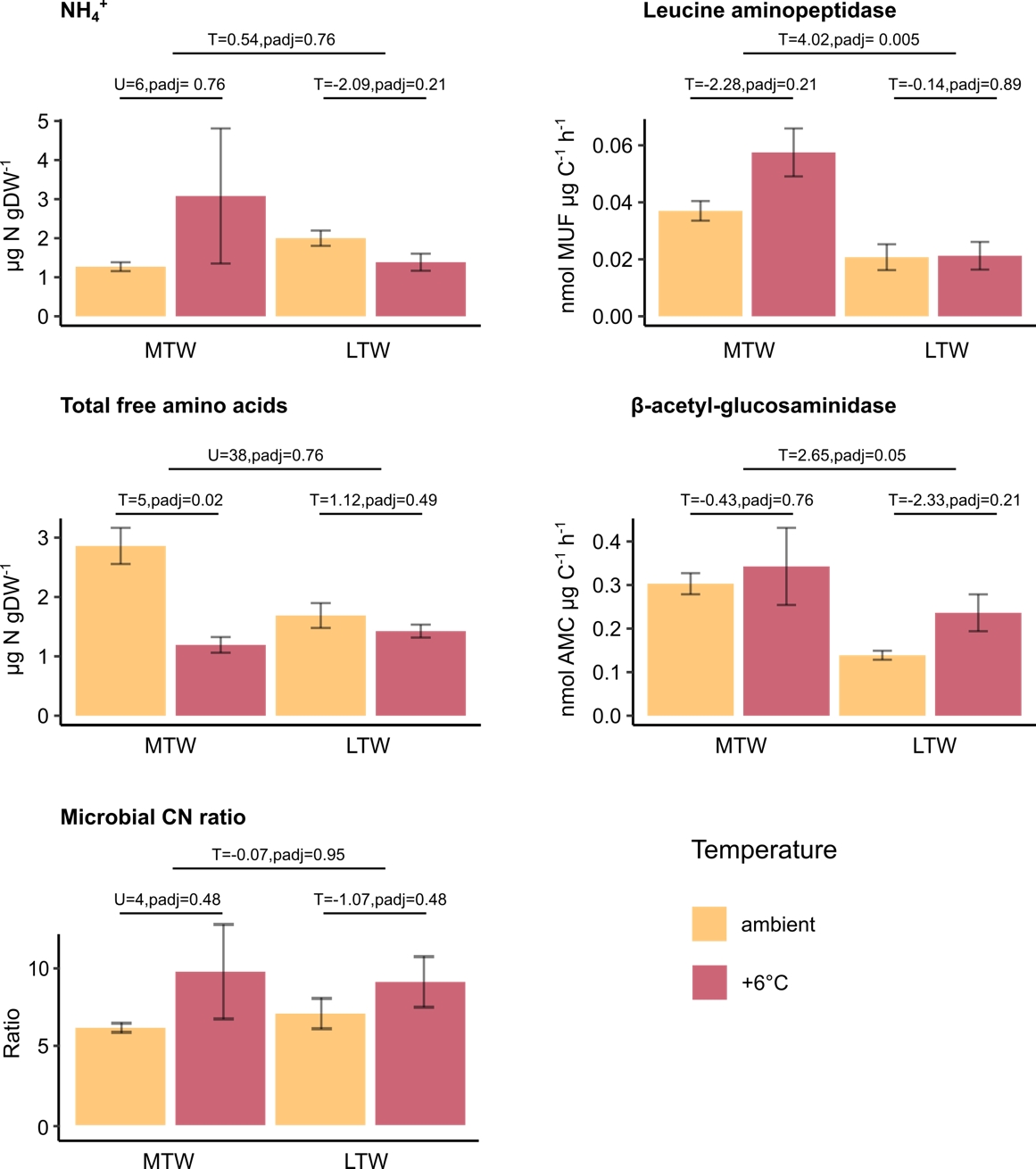


**Figure S4. Complementary data and potential enzymatic activities**

Complementary physicochemical data and potential enzymatic activities per unit of microbial biomass C are presented for the medium-term warmed (MTW) and long-term warmed (LTW) grasslands. Significant differences between groups and between temperature profiles were assessed using two-tailed parametric (T) and non-parametric (U) t-tests. Error bars depict the standard error from 4 biological replicates. Detailed statistics are presented in Table S7.

**Figure S5. Number of transcribed ON cycling genes per warming and grassland profiles**

The relative transcription levels of all genes encoding secreted proteins putatively involved in organic N degradation in relation to all protein coding genes in each sample are shown as transcripts per million (TPM) between ambient and warmed (+ 6 ºC) conditions (A), between warming durations (LTW, long-term warming; MTW, medium-term warming) (B). Significant differences between sites and between temperature profiles were assessed using two-tailed parametric (T) and non-parametric (U) t-tests. Detailed statistics are presented in Table S7.

**Figure S6.** Number of unique functions (A) and genes (B) associated with the degradation of organic N sources in the medium-term warmed (MTW) and in the long-term warmed (LTW) grasslands. Significant differences between sites and between temperature profiles were assessed using non-parametric (U) t-tests. Detailed statistics are presented in Table S7.


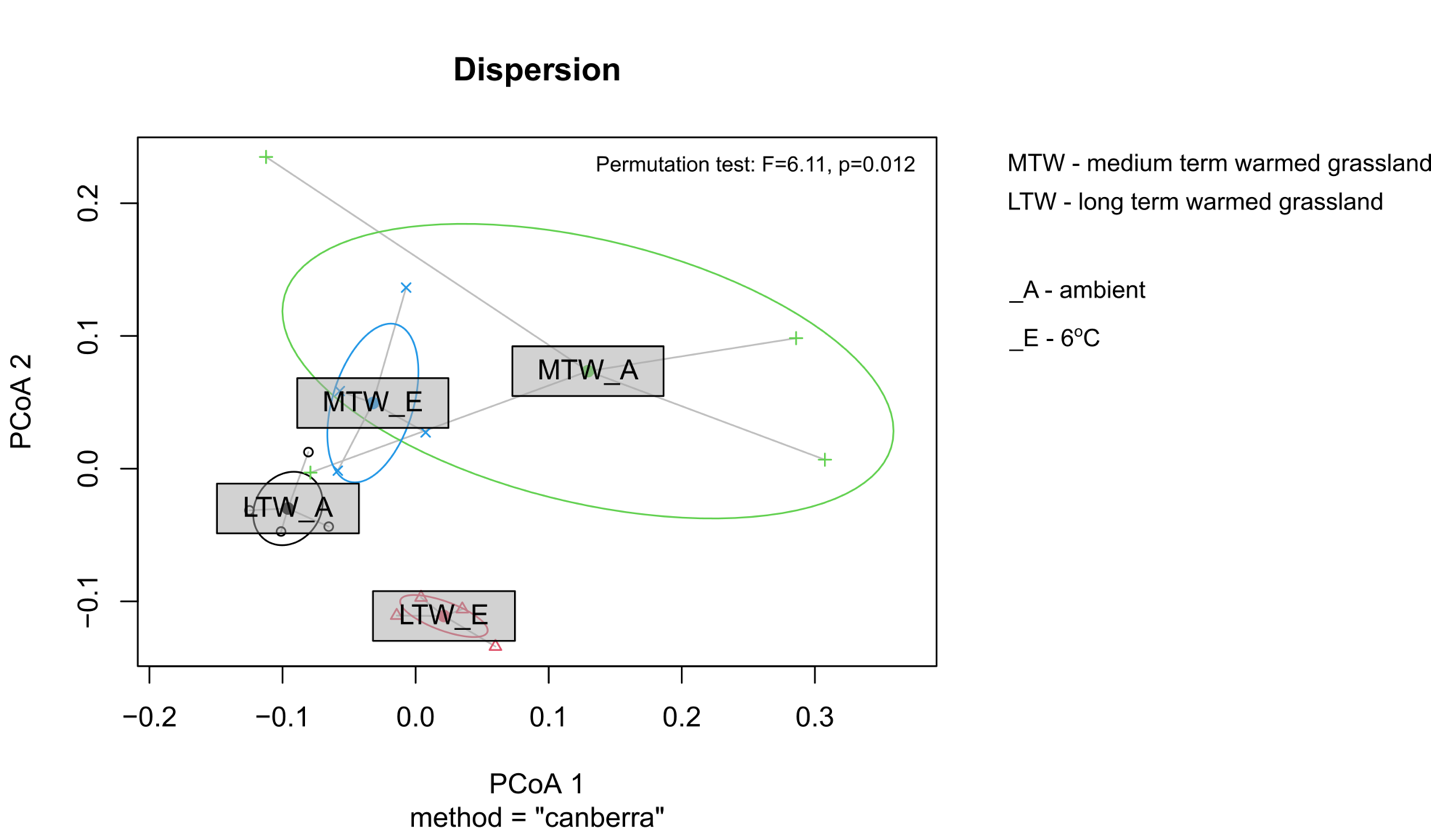


**Figure S7. Analysis of multivariate homogeneity of group dispersions (variances).**

Results from PERMDIST indicate significant differences in group dispersions, likely caused by the high dispersion from the MTW_A samples.

**Figure S8.** Taxonomic classification of all transcribed genes involved in the degradation of organic N sources. Phyla with a relative abundance higher than 0.01% are represented for the medium-term warmed (A) and long-term warmed (B) grasslands. Classes within phylum Proteobacteria are shown individually. The heatmaps show the relative abundance of each taxonomic group in the ambient and warmed plots for each grassland. The data were normalized by row (z-score) and clustered using complete linkage, according to similar distribution patterns. On the top of each heatmap, the percentage of variation explained by temperature is shown, as a result of a PERMANOVA analysis. The barplots show the combined abundance of each taxonomic group across all biological replicates for each temperature group.

**Figure S9.** Taxonomic classification of all transcribed genes involved in the degradation of chitin (A), nucleic acids (B), microbial cell walls (C) and proteins (D) in the long-term warmed grassland. Phyla with relative abundances above 0.01% are represented. Classes within phylum Proteobacteria are shown individually. The heatmaps show the relative abundance of each taxonomic group in the ambient and warmed plots for each grassland. The data were normalized by row (z-score) and clustered using complete linkage, according to similar distribution patterns. On the top of each heatmap, the percentage of variation explained by temperature is shown, as a result of a PERMANOVA analysis. The barplots show the combined abundance of each taxonomic group across all biological replicates for each temperature group.

Figure S10. Taxonomic classification of all transcribed genes involved in the degradation of chitin (A), nucleic acids (B), microbial cell walls (C) and proteins (D) in the medium-term warmed grassland. Phyla with relative abundances > 0.01% are represented. Classes within phylum Proteobacteria are shown individually. The heatmaps show the relative abundance of each taxonomic group in the ambient and warmed plots for each grassland. The data were normalized by row (z-score) and clustered using complete linkage, according to similar distribution patterns. On the top of each heatmap, the percentage of variation explained by temperature is shown, as a result of a PERMANOVA analysis. The barplots show the combined abundance of each taxonomic group across all biological replicates for each temperature group.

**Supplementary Tables**
